# Knockdown of long non-coding RNA HOTAIR reverses cisplatin resistance of ovarian cancer cells through inhibiting miR-138-5p-regulated EZH2 and SIRT1

**DOI:** 10.1186/s40659-020-00286-3

**Published:** 2020-04-29

**Authors:** Yun Zhang, Hao Ai, Xue Fan, Suxian Chen, Yadi Wang, Lili Liu

**Affiliations:** 1 Department of Obstetrics and Gynecology, The First Affiliated Hospital of Jinzhou Medical University, No. 2, Section 5, Renmin Street, Jinzhou, 121001 Liaoning People’s Republic of China; 2 Liaoning Key Laboratory of Follicular Development and Reproductive Health, Jinzhou, 121001 Liaoning People’s Republic of China; 3 Department of Obstetrics and Gynecology, The Third Affiliated Hospital of Jinzhou Medical University, Jinzhou, 121002 Liaoning People’s Republic of China; 4 Department of Pathology, The Third Affiliated Hospital of Jinzhou Medical University, Jinzhou, 121002 Liaoning People’s Republic of China; 5 Department of Oncology, The Third Affiliated Hospital of Jinzhou Medical University, Jinzhou, 121002 Liaoning People’s Republic of China

**Keywords:** Ovarian cancer, DDP resistance, HOTAIR, miR-138-5p

## Abstract

**Background:**

Cisplatin resistance (DDP-resistance) remains one of the major causes of poor prognosis in females with ovarian cancer. Long non-coding RNAs (lncRNAs) have been shown to participate in the regulation of cellular processes, including chemoresistance. The aim of this study was to explore the role of HOX transcript antisense RNA (HOTAIR) in DDP-resistant ovarian cancer cells.

**Methods:**

DDP-resistant ovarian cancer cell lines (SKOV3/DDP and A2780/DDP) were established. Real-time PCR, western blot, dual-luciferase reporter assay, and flow cytometry were then used to evaluate the effect of HOTAIR/miR-138-5p axis on chemoresistance of DDP-resistant ovarian cancer cells to DDP.

**Results:**

We found that HOTAIR was upregulated in DDP-resistant cells, while miR-138-5p was downregulated. Knockdown of HOTAIR increased the expression of miR-138-5p in DDP-resistant cells and miR-138-5p is directly bound to HOTAIR. Upregulation of miR-138-5p induced by HOTAIR siRNA or by its mimics enhanced the chemosensitivity of DDP-resistant cells and decreased the expression of EZH2 (enhancer of zeste 2 polycomb repressive complex 2 subunit) and SIRT1 (sirtuin 1). Furthermore, the HOTAIR silencing-induced chemosensitivity of DDP-resistant cells was weakened by miR-138-5p inhibitor.

**Conclusions:**

These data demonstrate that HOTAIR acts as a sponge of miR-138-5p to prevent its binding to EZH2 and SIRT1, thereby promoting DDP-resistance of ovarian cancer cells. Our work will shed light on the development of therapeutic strategies for ovarian cancer treatment.

## Background

Ovarian cancer, one of the most lethal diseases in the female reproductive system, is responsible for 4% of deaths from cancer in women [1]. Ovarian cancer can be divided into three broad subgroups: epithelial, stromal, and germ cell tumors, of which epithelial ovarian cancer is the most lethal type of ovarian cancer and accounts for 85% of all reported cases [2]. Cisplatin (DDP) is one of the first line agents employed in the treatment of epithelial ovarian cancer [3]. However, DDP-resistance is frequently observed in advanced epithelial ovarian cancer patients and predicts poor prognosis [4]. Therefore, it is important to investigate the molecular basis of DDP-resistance in ovarian cancer and identify more effective therapeutic strategies.

Long non-coding RNAs (lncRNAs), a class of non-coding transcripts, have recently been reported as important regulators of cell proliferation, invasion, and apoptosis in several cancer types [5–7]. Moreover, multiple lines of evidences showed that lncRNAs were dysregulated in various types of cancers [8–10]. The HOX transcript antisense RNA (HOTAIR) gene has been identified and located within the Homeobox C (HOXC) gene cluster on Chromosome 12 and encodes a 2.2 kb lncRNA molecule [11]. HOTAIR expression was initially found to be upregulated in primary breast tumors and metastases [12]. In recent studies, upregulation of HOTAIR has been proven to be associated with the metastasis of various malignant tumors, such as colorectal cancer [13], hepatocellular carcinoma [8], and pancreatic carcinoma [14]. Moreover, a relatively small number of studies have associated HOTAIR with ovarian cancer. Although recent studies found that overexpression of HOTAIR could lead to chemoresistance in ovarian cancer [15, 16], the underlying molecular mechanism needs to be further investigated.

MicroRNA-138-5p (miR-138-5p), a non-coding small RNA molecule which only expressed in the ovaries, was recently identified as a cancer suppressor by post-transcriptionally repressing the expression of proto-oncogenes [17–19]. Unfortunately, although the potential effectiveness was identified in hepatocellular carcinoma [20], non-small cell lung cancer [21] and nasopharyngeal carcinoma [22], the role of miR-138-5p involved in DDP resistance of ovarian cancer cells needs to be addressed. Moreover, there is no report about the correlation between HOTAIR and miR-138-5p on regulating DDP resistance in ovarian cancer cells.

In this study, we detected the expression of HOTAIR and miR-138-5p in DDP-resistant cells and investigated correlation effects of HOTAIR and miR-138-5p in DDP resistant ovarian cancer cells.

## Materials and methods

### Cell culture and transfection

Two ovarian cancer cell lines, SKOV3 and A2780 were purchased from Procell Life Science &Technology Co., Ltd. (Wuhan, China), cultured in Dulbecco’s modified Eagle’s medium (Sigma, St. Louis, MO, USA) containing 10% fetal bovine serum (Sigma), and maintained at 37 °C with 5% CO_2_. Drug-resistant cell lines of SKOV3 and A2780 were constructed by treatment of proliferating cell cultures with DDP (Dalian Meilun Biotechnology Co., Ltd., Dalian, China) at final concentrations of 8 µM for 12 weeks.

Drug-resistant cells were seeded into 6-well plates and transfected with miR-138-5p mimic, negative control (NC) mimic (a non-specific miRNA mimic), miR-138-5p inhibitor, NC inhibitor (a non-targeting miRNA inhibitor), si-HOTAIRs, or NC siRNA (a non-targeting siRNA) using Lipofectamine 2000 (Invitrogen, Carlsbad, CA, USA) according to the manufacturer’s protocols. The sequences were listed in Table 1. After 48 h post-transfection, drug-resistant cells were harvested for further analyses.

**Table 1:** The sequences of interference vectors

Name	Sequence
si-HOTAIR-1	5′-UAACAAGACCAGAGAGCUGTT-3′
	5′-CAGCUCUCUGGUCUUGUUATT-3′
si-HOTAIR-2	5′-GAACGGGAGUACAGAGAGATT-3′
	5′-UCUCUCUGUACUCCCGUUCTT-3′
NC siRNA	5′-UUCUCCGAACGUGUCACGUTT-3′
	5′-ACGUGACACGUUCGGAGAATT-3′
mir-138-5p mimics	5′-AGCUGGUGUUGUGAAUCAGGCCG-3′
	5′-GCCUGAUUCACAACACCAGCUUU-3′
NC mimics	5′-UUCUCCGAACGUGUCACGUTT-3′
	5′-ACGUGACACGUUCGGAGAATT-3′
miR-138-5p inhibitor	5′-CGGCCUGAUUCACAACACCAGCU-3′
NC inhibitor	5′-CAGUACUUUUGUGUAGUACAA-3′

### RNA extraction and real-time PCR analysis

Total RNAs were extracted from cells with the TRIpure reagent (BioTeke, Beijing, China) according to the manufacturer’s instructions. Then cDNAs were synthesized using Super M-MLV reverse transcriptase (BioTeke). Real-time PCR reactions were performed in a mixture of 20 µl consisting 1 µl cDNA, 0.5 μl of 10 μM forward/reverse primer, 0.3 µl of SYBR Green, and 10 μl of 2 × Power Taq PCR using an Exicycler 96 Real-Time Quantitative Thermal Block (Bioneer, Korea). Data were collected and analyzed by the 2^−∆∆CT^ method. The sequences of primers used for PCR were listed in Table 2. We used beta-actin as an internal reference for HOTAIR detection based on the geNorm analysis [23]. U6 was used as an internal control for miR-138-5p detection.

**Table 2:** The sequences of primers used for PCR

Name	Sequence
HOTAIR F	5′-TAGGCAAATGTCAGAGGGTT-3′
HOTAIR R	5′-CTTAAATTGGGCTGGGTCT-3′
β-actin F	5′-ACCCTGAAGTACCCCATCGA-3′
β-actin R	5′-CAAACATGATCTGGGTCATCT-3′
hsa-miR-138-5p F	5′-GCCGAGCTGGTGTTGTGAAT-3′
hsa-miR-138-5p R	5′-GTGCAGGGTCCGAGGTATTC-3′
U6 F	5′-GCTTCGGCAGCACATATACT-3′
U6 R	5′-GTGCAGGGTCCGAGGTATTC-3′

### MTT assay

Cells (5 × 10^3^ cells per well) were seeded in 96-well plates. DDP with a range of concentrations (0 μM, 5 μM, 10 μM, 20 μM, 40 μM, 80 μM) was added into each group. The plates were then incubated at 37 °C for an additional 48 h. Thereafter, the culture solutions were replaced by new medium with MTT (0.5 mg/ml) and the plates were then incubated for 4.5 h at 37 °C with 5% CO_2_. After that, the culture solutions were carefully removed and 150 µl of DMSO was added into each well to completely dissolve the generated formazan crystals. The absorbance was measured at a wavelength of 570 nm using a microplate absorbance reader (BioTek, Winooski, VT, USA).

### Western blot

Cells were lysed with RIPA lysis buffer (Beyotime Institute of Biotechnology, Shanghai, China) to obtain total proteins. Protein concentration was determined using the BCA Protein Assay Kit (Beyotime Institute of Biotechnology). Protein samples (20–40 µg) were subjected to sodium dodecyl sulfate–polyacrylamide gel electrophoresis (SDS-PAGE), transferred to polyvinylidene fluoride (PVDF) membranes (Millipore, Massachusetts, USA), and blocked with 5% skimmed milk. After that, the membrane was treated with primary antibodies against Enhancer of zeste 2 polycomb repressive complex 2 subunit (EZH2; 1:1000, CST, Danvers, MA, USA), sirtuin 1 (SIRT1; 1:1000, CST), Bcl2 (1:500, Beyotime Institute of Biotechnology), Bax (1:500, Beyotime Institute of Biotechnology), cleaved caspase 3 (CI-caspase 3; 1:000, Beyotime Institute of Biotechnology), cleaved PARP (CI-PARP; 1:000, Beyotime Institute of Biotechnology), and β-actin (1:1000, Santa Cruz Biotechnology, Santa Cruz, CA, USA) overnight at 4 °C, followed by incubation of horseradish peroxidase-conjugated goat anti-rabbit or anti-mouse secondary antibodies (1:5000, Beyotime Institute of Biotechnology) for 45 min at 37 °C. Then, enhanced chemiluminescence (ECL) chromogenic substrates were used for protein visualization with a gel imaging system (Beijing Liuyi Instrument Factory, Beijing, China), and the protein bands were quantified by optical densitometry (Gel-Pro-Analyzer software).

### Apoptosis assay

Apoptosis was measured using an Annexin V/FITC and propidium iodide (PI) apoptosis detection kit (Keygen, Nanjing, China). Briefly, collected cells were gently resuspended in 500 μl of binding buffer. Then, cells were incubated with 5 μl Annexin V-FITC and 5 μl PI for 10 min away from light. The cells were analyzed by a flow cytometer (Aceabio, San Diego, CA, USA).

### Luciferase assay

Wild type fragments of HOTAIR, EZH2 3′UTR and SIRT1 3′UTR containing putative binding sites for miR-138-5p or mutant-type fragments (putative binding sites for miR-138-5p were mutated) were cloned into the pmirGLO vector between SalI and NheI restriction enzyme sites. The constructed plasmids and miR-138-5p mimic were co-transfected into 293T cells for 48 h using Lipofectamine 2000 (Invitrogen). Firefly and Renilla luciferase activities were determined using the dual luciferase assay system (Promega, Madison, WI, USA), and firefly luciferase activity was normalized to Renilla luciferase activity, according to the manufacturer’s directions.

### Statistical analysis

Data are presented as means ± standard errors. Student’s t-test and one-way analysis of variance were used to determine the significance between groups. Statistical analysis was carried out using Graphpad Prism 7. Differences with p-values of < 0.05 were considered statistically significant.

## Results

### Expression levels of HOTAIR and miR-138-5p in DDP-resistant ovarian cancer cells

**Fig. 1:**
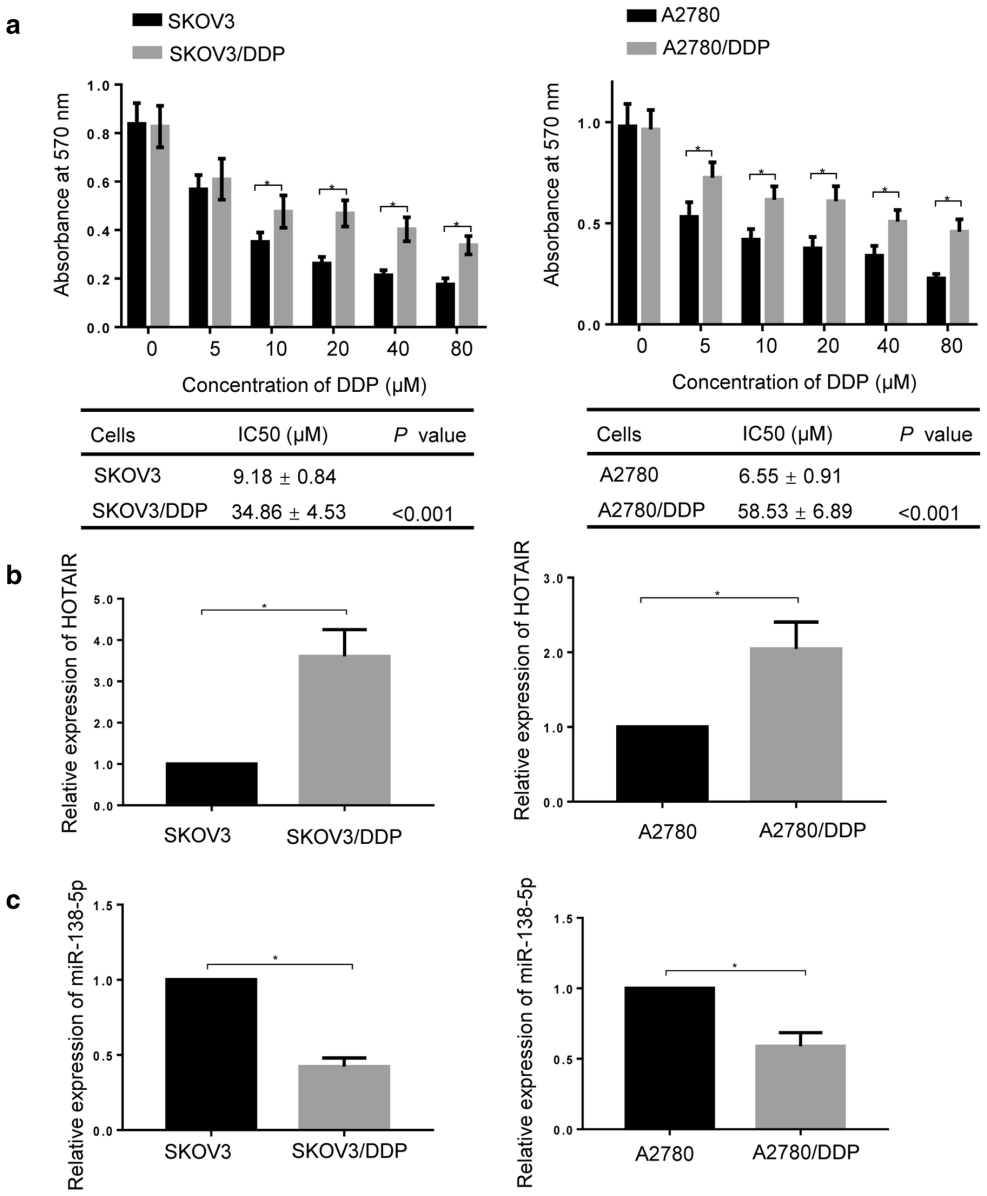
Expression levels of HOTAIR and miR-138-5p in DDP-resistant ovarian cancer cells. **a** The effect of different concentrations of DDP on cell viability in DDP sensitive (SKOV3 and A2780) and DDP resistant (SKOV3/DDP and A2780/DDP) cells was determined by MTT assay, and the IC50 value was obtained. Real-time PCR was employed to evaluate the expression of **b** HOTAIR and **c** mir-138-5p in DDP sensitive (SKOV3 and A2780) and DDP resistant (SKOV3/DDP and A2780/DDP) cells, beta-actin and U6 were used as an internal control for HOTAIR and miR-138-5p detection, respectively. Data were presented as mean ± SD. * *p* < 0.05

In order to investigate the effect of HOTAIR/miR-138-5p axis on DDP-resistance in ovarian cancer, two cisplatin chemo-resistant ovarian cell lines (SKOV3/DDP and A2780/DDP) were constructed by treatment of SKOV3 and A2780 cells (the two mostly commonly used cellular models of ovarian cancer) with DDP (8 µM) for 12 weeks. The half-maximal inhibitory concentration (IC_50_) values of both DDP-sensitive (SKOV3 and A2780) and DDP-resistant (SKOV3/DDP and A2780/DDP) cells were determined by MTT assay. As shown in Fig. 1a, SKOV3/DDP and A2780/DDP cells performed significantly enhanced DDP-resistance (*p < 0.05). The calculated IC50 values of SKOV3 cell line increased significantly from 9.18 ± 0.84 μM to 34.86 ± 4.53 μM (*p < 0.001), and the IC_50_ values of A2780 cell line increased remarkably from 6.55 ± 0.91 μM to 58.53 ± 6.89 μM (*p < 0.001). Furthermore, the expression of HOTAIR and miR-138-5p in DDP-resistant cells was detected by real-time PCR. As shown in Fig. 1b, the expression levels of HOTAIR were increased in both SKOV3/DDP and A2780/DDP cell lines (*p < 0.05). Inversely, the expression levels of miR-138-5p were decreased in DDP-resistant cell lines (*p < 0.05) (Fig. 1c). The results suggested that HOTAIR and miR-138-5p might be implicated in the development of DDP resistance of ovarian cancer cells. Moreover, the negative correlation between the expression of HOTAIR and mir-138-5p might indicate potential binding in DDP-resistant ovarian cancer cells

### HOTAIR targets and modulates the expression of miR-138-5p

**Fig. 2:**
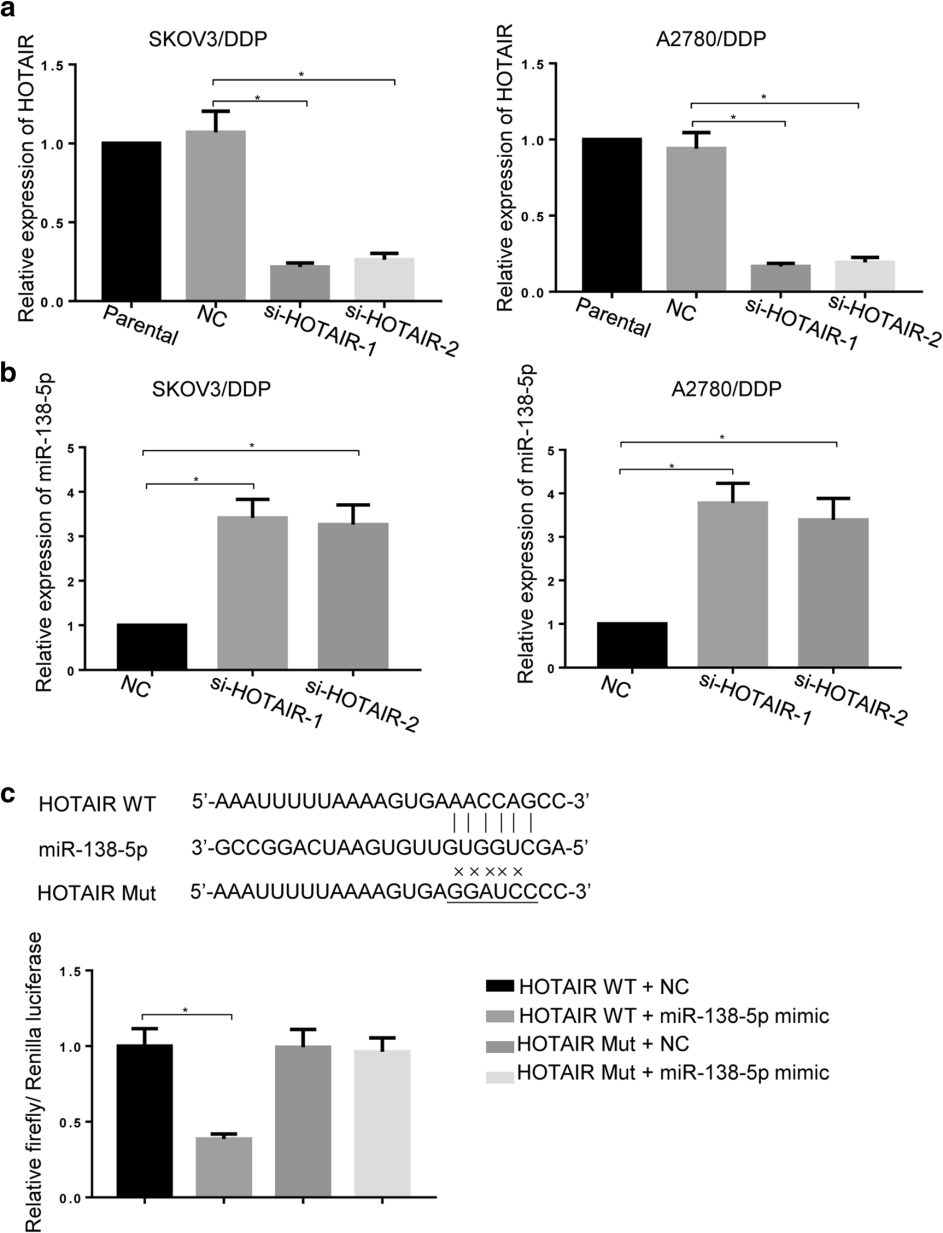
Knockdown of HOTAIR increased miR-138-5p expression in DDP-resistant ovarian cancer cells. **a** Downregulation of HOTAIR by two different small interfering RNAs. **b** The expression of miR-138-5p in DDP-resistant ovarian cancer cells after silencing of HOTAIR was assessed by real-time PCR, beta-actin and U6 were used as an internal control for HOTAIR and miR-138-5p detection, respectively. **c** Dual-luciferase assay was used to verify the direct binding of miR-138-5p and HOTAIR. Data were presented as mean ± SD. * *p* < 0.05

The relationship between HOTAIR and miR-138-5p expression was further explored. Two ovarian cancer DDP-resistant cell lines (SKOV3/DDP and A2780/DDP) were transfected with designed HOTAIR small interfering RNA fragments (si-HOTAIR1 and si-HOTAIR2) to silence the expression of HOTAIR. The effectiveness was verified by real-time PCR experiments (Fig. 2a). Compared with negative controls, knockdown of HOTAIR dramatically increased the expression of miR-138-5p (Fig. 2b) (*p < 0.05). Bioinformatics prediction for potential binding sites between HOTAIR and miR-138-5p was shown in Fig. 2c. Moreover, the relative luciferase activities in cells co-transfected with HOTAIR WT and miR-138-5p mimic (which is a double-stranded RNA consisting of the guide strand that is designed to mimic the function of the endogenous miRNA and the passenger strand that is partially complementary to the guide strand) was significantly downregulated (*p < 0.05), indicating that HOTAIR can bind to miR-138-5p (Fig. 2c).

### MiR-138-5p positively regulates DDP sensitivity of ovarian cancer cells

**Fig. 3:**
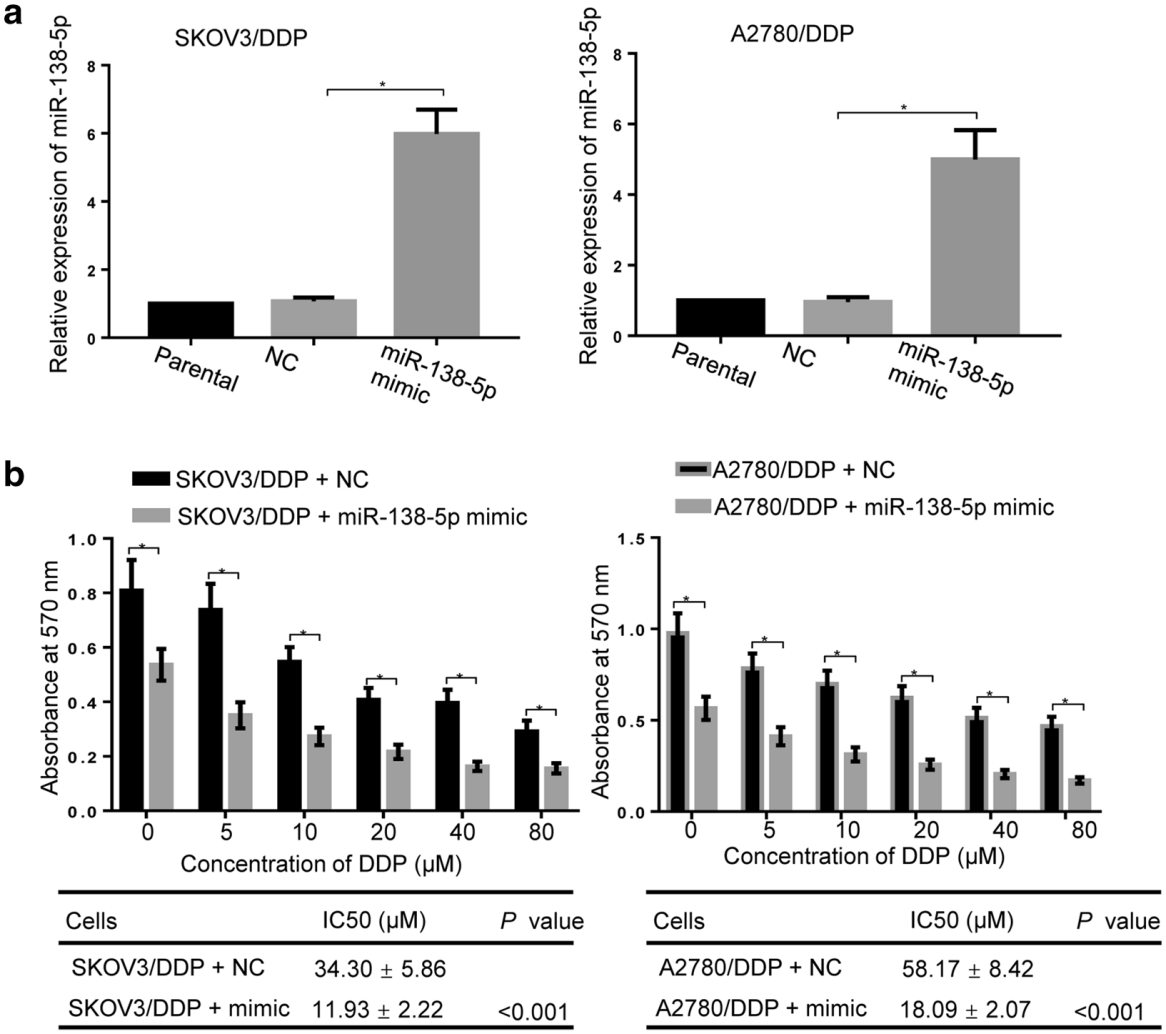
Upregulation of miR-138-5p enhanced chemosensitivity of DDP-resistant ovarian cancer cells to DDP. **a** Verification of overexpression of miR-138-5p by real-time PCR assay, U6 were used as an internal control. **b** The effect of different concentrations of DDP on cell viability in SKOV3/DDP and A2780/DDP cells with or without forced overexpression of miR-138-5p was assessed by MTT assay, and the IC50 value was obtained. Data were presented as mean ± SD. * *p* < 0.05

SKOV3/DDP and A2780/DDP cells were transfected with miR-138-5p mimic or negative control, and transfection efficiency was detected after 48 h by real-time PCR. As shown in Fig. 3a, the relative expression of miR-138-5p in both SKOV3/DDP and A2780/DDP cells was significantly increased following miR-138-5p mimic treatment (*p < 0.05). After that, the influence of DDP on cell viability in SKOV3/DDP and A2780/DDP cells with or without forced overexpression of miR-138-5p was measured by MTT assay. As shown in Fig. 3b, transfection with the miR-138-5p mimic remarkably inhibited cell proliferation compared to transfection with the negative control (*p < 0.05). The calculated IC_50_ values of transfected SKOV3/DDP cell line decreased significantly from 34.30 ± 5.86 μM to 11.93 ± 2.22 μM (*p < 0.001), and the IC_50_ values of A2780/DDP cell line decreased significantly from 58.17 ± 8.42 μM to 18.09 ± 2.07 μM (*p < 0.001).

**Fig. 4:**
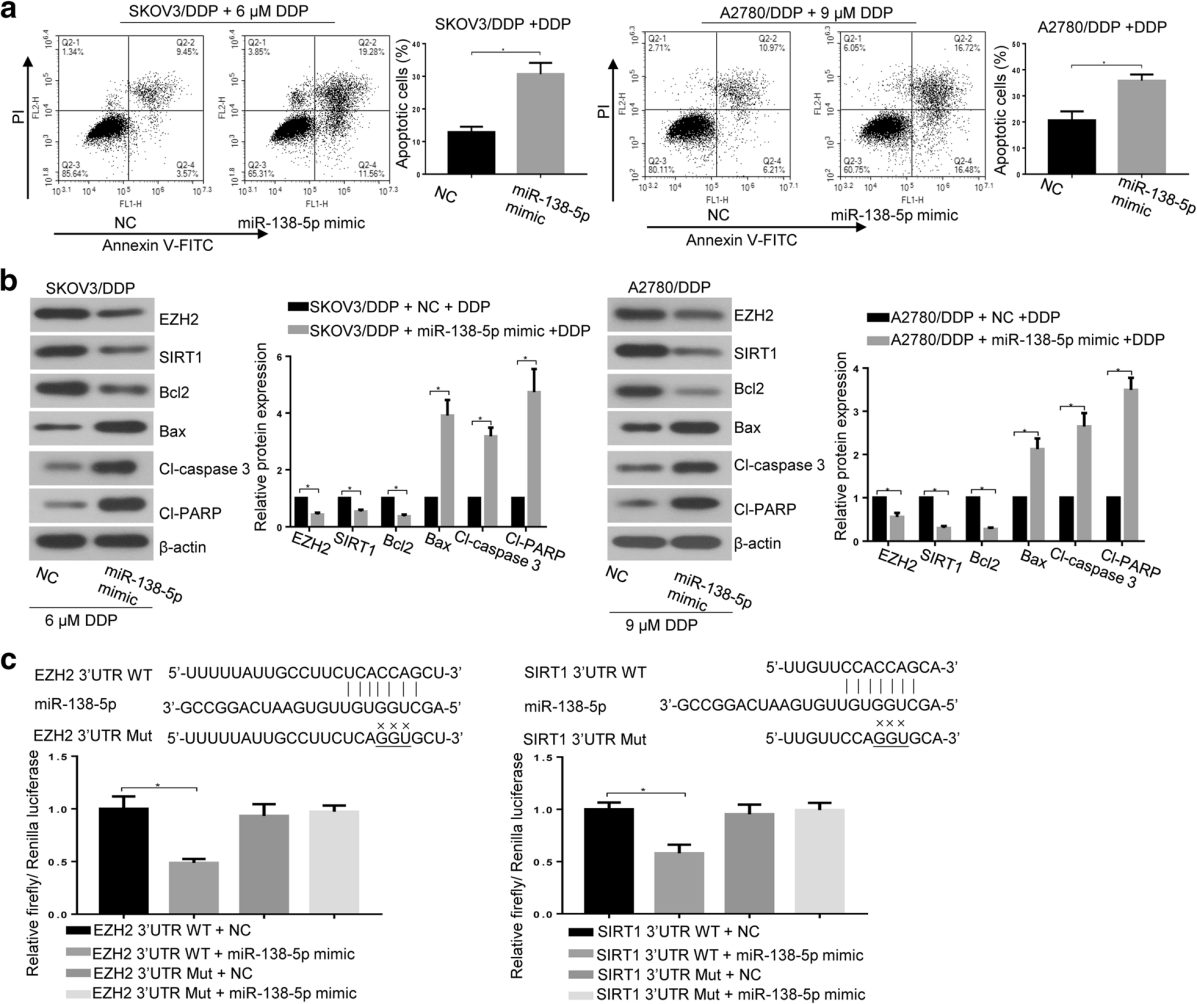
Upregulation of miR-138-5p promoted DDP-mediated apoptosis in DDP-resistant ovarian cancer cells. **a** Cell apoptosis in DDP-treated SKOV3/DDP and A2780/DDP cells with miR-138-5p overexpression was determined by annexin V-FITC and PI staining through flow cytometry. **b** Expression levels of EZH2, SIRT1, Bcl2, Bax, Cl-caspase 3 and Cl-PARP were evaluated by western blot analysis. **c** Dual-luciferase assay was used to verify the direct binding of miR-138-5p with EZH2 and SIRT1. Data were presented as mean ± SD. * *p* < 0.05

To further explore whether miR-138-5p positively regulated cell apoptosis of DDP-resistant ovarian cancer cells, SKOV3/DDP and A2780/DDP cells were transfected with miR-138-5p mimic, followed by DDP treatment (6 μM for SKOV3/DDP and 9 μM for A2780/DDP). As shown in Fig. 4a, flow cytometry analysis revealed that the increase in apoptotic rate was evidently proved after overexpressing miR-138-5p in SKOV3/DDP and A2780/DDP cells (*p < 0.05). Moreover, western blot analysis showed that the protein levels of EZH2, SIRT1 and Bcl2 were markedly decreased in DDP-treated drug-resistant cells following miR-138-5p upregulation, while protein expression levels of Bax, Cl-caspase 3 and Cl-PARP were increased (Fig. 4b) (*p < 0.05). The dual-luciferase assay showed that miR-138-5p could directly bind to the 3′ UTR of EZH2 and SIRT1 (Fig. 4c) (*p < 0.05). Taken together, the results indicated that the forced expression of miR-138-5p increased the cisplatin chemosensitivity.

### Downregulation of HOTAIR contributes to DDP-sensitivity of ovarian cancer cells by upregulation of miR-138-5p

**Fig. 5:**
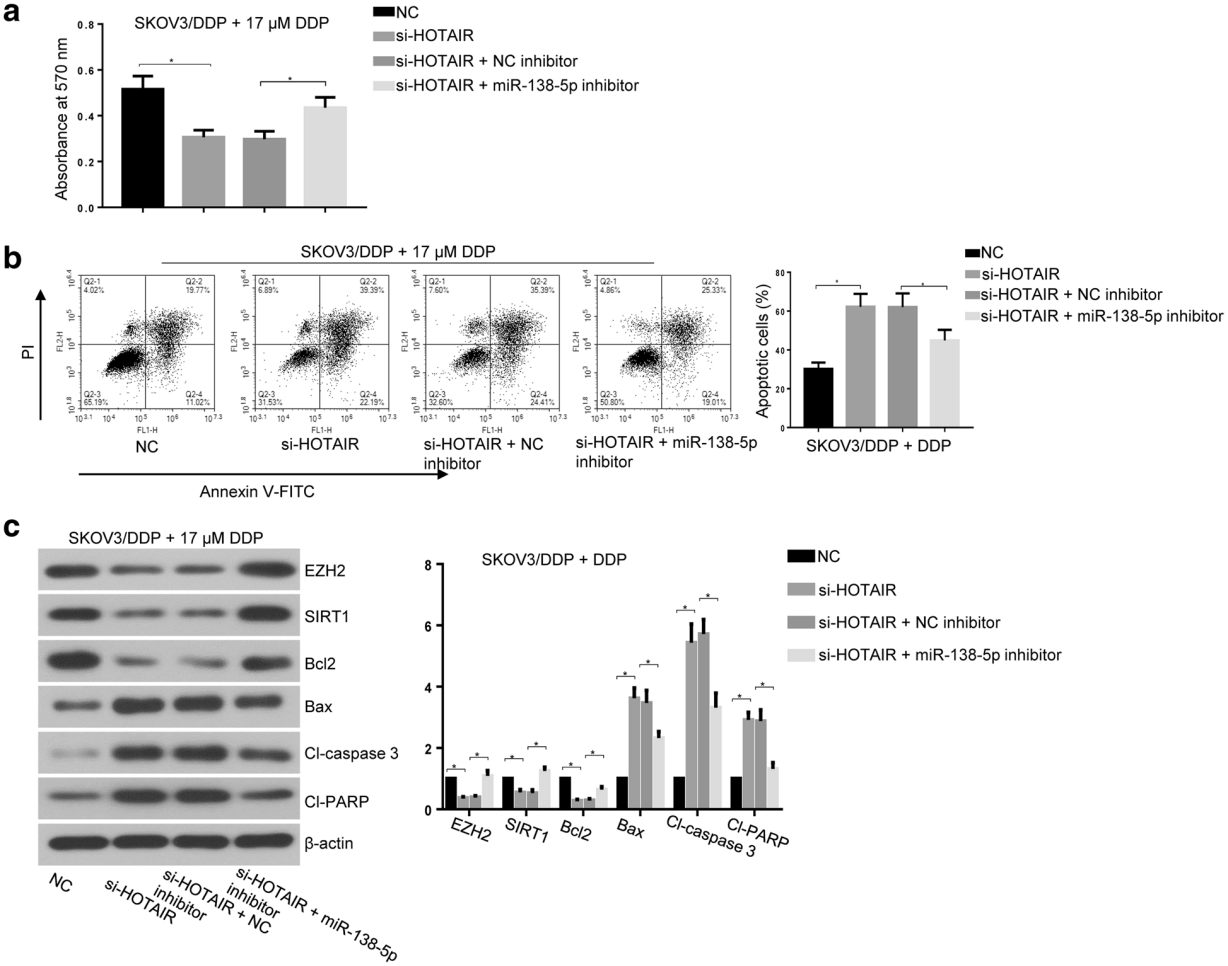
Downregulation of miR-138-5p reversed silencing of HOTAIR mediated pro-apoptosis activity in DDP-treated SKOV3/DDP cells. **a** Cell viability in DDP-treated SKOV3/DDP cells with HOTAIR silencing or/and miR-138-5p silencing was determined by MTT assay. **b** Cell apoptosis was assessed by annexin V-FITC and PI staining with flow cytometry. **c** Expression levels of EZH2, SIRT1, Bcl2, Bax, Cl-caspase 3 and Cl-PARP were assessed by western blot assay. Data were presented as mean ± SD. * *p* < 0.05

Knockdown of HOTAIR inhibited cell viability and promoted cell apoptosis in DDP-treated SKOV3/DDP cells (Fig. 5a, b), which was accompanied by the decrease of EZH2, SIRT1 and Bcl2 and by the increase of Bax, Cl-caspase 3 and Cl-PARP (Fig. 5c). Furthermore, silencing of miR-138-5p reversed the effect of HOTAIR knockdown on chemosensitivity of SKOV3/DDP cells to DDP (Fig. 5a–c). Overall, these data suggested that HOTAIR modulates cisplatin chemosensitivity of ovarian cancer cells by miR-138-5p.

## Discussion

Ovarian cancer is the leading cause of death in gynecologic cancers, and DDP-based chemotherapy is one of the first-line therapeutic approaches for ovarian cancer [24, 25]. However, chemotherapy resistance strikingly decreased therapeutic efficiency and contributed to a high relapse rate in ovarian cancer [26]. Therefore, it is of importance to illuminate the molecular mechanism underlying the DDP-resistance for improvement of the prognosis.

LncRNAs can function as a competing endogenous RNA (ceRNA) by sponging miRNAs to regulate gene expression at the post-transcriptional level. HOTAIR regulates HER2 expression by sponging miR-331-3p in gastric cancer [27]. HOTAIR regulates CCND1 and CCND2 expression by sponging miR-206 in ovarian cancer [28]. Previous studies have been reported that HOTAIR was negatively correlated with DDP-resistance in gastric cancer [29–31], human lung adenocarcinoma [32], and small cell lung cancer [33]. For instance, HOTAIR was considered to play an important role in gastric cancer by PI3K/Akt and Wnt/β-catenin signaling pathways by up-regulating miR-34a. Knockdown of HOTAIR increased the inhibitory effect of DDP on tumor growth in vivo [29]. However, the molecular mechanism of HOTAIR in DDP resistance of ovarian cancer remains undefined.

Clinical studies demonstrated that the expression levels of miR-138-5p were significantly upregulated in patients with ovarian cancer [34]. Moreover, Chen et al. [18] indicated that miR-138 could directly target LIM kinase 1 (Limk1) and inhibited metastasis of ovarian cancer by Limk1/cofilin/p-cofilin signaling pathway. Yeh et al. [35] pointed out that miR-138 regulated ovarian cancer cell invasion and metastasis by targeting SOX4 and HIF-1a oncogenic transcriptional factors. Unfortunately, the roles of miR-138-5p in DDP-resistance in ovarian cancer cells remain unclear. In this study, we successfully obtained DDP-resistant ovarian cancer cells (SKOV3/DDP and A2780/DDP). Increased HOTAIR and decreased miR-138-5p expression were found in SKOV3/DDP and A2780/DDP cells. Knockdown of HOTAIR could increase the expression of miR-138-5p, which could enhance the chemosensitivity of DDP-resistance cells to DDP. Moreover, we demonstrated that miR-138-5p could directly regulate the expression of EZH2 and SIRT1, which have been proven to be involved in regulation of cisplatin resistance of ovarian cancer [36, 37]. Interestingly, our data showed that downregulation of miR-138-5p only partially reversed the HOTAIR silencing-mediated pro-apoptosis activity, suggesting that other miRNAs or molecules may also contribute to the action of HOTAIR on chemosensitivity of ovarian cancer cells to DDP. Many miRNAs (such as miR-34a and miR-454) reported to be involved in regulating the chemosensitivity of ovarian cancer were regulated by HOTAIR [38–41], indicating that HOTAIR may also mediate chemoresistance of ovarian cancer cells by other miRNAs and their target genes. Moreover, the efficacy of HOTAIR/miR-138-5p axis on the treatment in clinical studies needs further explorations.

## Conclusion

In the present study, we demonstrated that HOTAIR/miR-138-5p axis could regulate the DDP-resistance of ovarian cancer by potential targets EZH2 and SIRT1, which could shed light on new therapeutic targets for ovarian cancer treatment.

## Data Availability

The datasets used and/or analyzed during the current study are available from the corresponding author on reasonable request.

## Abbreviations

DDP: Cisplatin
LncRNA: Long non-coding RNA
HOTAIR: HOX transcript antisense RNA
EZH2: Enhancer of zeste 2 polycomb repressive complex 2 subunit
SIRT1: Sirtuin 1
miR-138-5p: MicroRNA-138-5p
